# Effect of early and current *Helicobacter pylori* infection on the risk of anaemia in 6.5-year-old Ethiopian children

**DOI:** 10.1186/s12879-015-1012-y

**Published:** 2015-07-14

**Authors:** Bineyam Taye, Fikre Enquselassie, Aster Tsegaye, Alemayehu Amberbir, Girmay Medhin, Andrew Fogarty, Karen Robinson, Gail Davey

**Affiliations:** School of Public Health, College of Health Sciences, Addis Ababa University, PO Box 80596, Addis Ababa, Ethiopia; School of Allied Health Sciences, College of Health Sciences, Addis Ababa University, Addis Ababa, Ethiopia; Department of Infectious Disease Epidemiology, London School of Hygiene and Tropical Medicine, Nottingham, UK; Aklilu Lemma Institute of Pathobiology, Addis Ababa University, Addis Ababa, Ethiopia; Division of Epidemiology and Public Health, University of Nottingham, Nottingham, UK; Nottingham Digestive Diseases Biomedical Research Unit, School of Medicine, University of Nottingham, Nottingham, UK; Brighton & Sussex Medical School, Nottingham, UK

**Keywords:** *Helicobacter pylori*, Anaemia, Red cell indices, Birth cohort, Ethiopia

## Abstract

**Background:**

Epidemiological and clinical studies in high income countries have suggested that *Helicobacter pylori (H. pylori)* may cause anaemia, but evidence is lacking from low income countries.We examined associations between *H. pylori* infection in early childhood and anaemia at the age of 6.5 years in an Ethiopian birth cohort.

**Methods:**

In 2011/12, 856 children (85.1 % of the 1006 original singletons in a population-based birth cohort) were followed up at age six and half. An interviewer-led questionnaire administered to mothers provided information on demographic and lifestyle variables. Haemoglobin level and red cell indices were examined using an automated haematological analyzer (Cell Dyn 1800, Abbott, USA), and stool samples analyzed for *H. pylori* antigen. The independent effects of *H. pylori* infection (measured at age 3.5 and 6.5 years) on anaemia, haemoglobin level, and red cell indices (measured at age 6.5 years) were determined using multiple logistic and linear regression.

**Results:**

The prevalence of anemia was 34.8 % (257/739), and the mean (SD) haemoglobin concentration was 11.8 (1.1) gm/dl. Current *H. pylori* infection at age 6.5 years was positively, though not significantly related to prevalence of anaemia (adjusted OR, 95 % CI, 1.15; 0.69, 1.93, p = 0.59). Any *H. pylori* infection up to age 6.5 years was significantly associated with an increased risk of anaemia at age 6.5 (adjusted OR, 95 % CI, 1.68; 1.22, 2.32, p = 0.01). A significant reduction in haemoglobin concentration and red cell indices was also observed among children who had any *H. pylori* infection up to age 6.5 (Hb adjusted β = −0.19, 95 % CI, −0.35 to −0.03, p = 0.01; MCV adjusted β = −2.22, 95 % CI, −3.43 to −1.01, p = 0.01; MCH adjusted β = −0.63, 95 % CI, −1.15 to - 0.12, p = 0.01; and MCHC adjusted β = −0.67, 95 % CI, −1.21 to −0.14, p = 0.01), respectively.

**Conclusion:**

This study provides further evidence from a low income country that any *H. pylori* infection up to age 6.5 is associated with higher prevalence of anaemia, and reduction of haemoglobin level and red cell indices at age 6.5.

**Electronic supplementary material:**

The online version of this article (doi:10.1186/s12879-015-1012-y) contains supplementary material, which is available to authorized users.

## Background

*Helicobacter pylori (H. pylori),* a spiral-shaped pathogenic bacterium found on the human gastric mucosa, was first isolated by Warren and Marshall in 1982 [1]. The bacterium is a ubiquitous micro-organism, infecting half of the world's population [2] and is widely accepted as the main etiological factor for peptic ulcer disease and gastric malignancy [3, 4]. The infection is usually acquired in childhood and persists throughout life, causing disease mainly in adults [5, 6]. In children, however, the consequences of *H. pylori* infection in gastroduodenal diseases are not well understood [7]; the majority have no specific symptoms [8, 9] and peptic ulcer disease is relatively rare in childhood [10]. More recently however, there is growing interest in investigating the effects of *H. pylori* in extra-gastroduodenal diseases [8, 11]. In particular, evidence for an association between *H. pylori* and anaemia has gained support from a range of epidemiological studies [12–15]. Moreover, improvement in iron deficiency anaemia (IDA) after *H. pylori* eradication has been demonstrated in children and adults with unexplained IDA [16–18].

The mechanisms by which *H. pylori* infection can cause iron deficiency anaemia are still unclear [19], but plausible mechanisms have been proposed. One hypothesis that has attracted attention is that chronic *H. pylori* infection leads to atrophy of the gastric glands and reduction of gastric hydrochloric and ascorbic acid secretion [20]. This in turn leads to increases in intragastric pH, which may impair iron absorption [21]. Other possible mechanisms for IDA in *H. pylori*-infected subjects involve altered expression of iron transport regulators, and uptake of iron by *H. pylori* bacteria in the gastric mucosa [22, 23]. Lactoferrin is an iron-binding glycoprotein that is found in body fluids, and its secretion in the gastric mucosa seems to be influenced by some signal from *H. pylori*[22]. It appears that *H. pylori* then absorbs the iron from lactoferrin via a specific lactoferrin-binding protein that is expressed by *H. pylori* [20]. Lactoferrin levels in the gastric mucosa have been shown to be significantly higher in *H. pylori-*positive patients with IDA than those who are non-anaemic *H. pylori*-negative, non-anaemic *H. pylori*-positive, and *H. pylori*-negative with IDA [22].

Whilst there is a growing body of epidemiological evidence suggesting that *H. pylori* infection is associated with an increased risk of anaemia and iron deficiency, most studies to date are in adult high-income country populations and lack data in children from low income countries, though *H. pylori* is a very common bacterial infection, infecting more than 50 % of children in low income countries [24, 25].

In low income countries, well-documented causes of anaemia in children include dietary iron deficiency, vitamin A deficiency, and infectious diseases such as malaria and hookworm [26], but the potential role of *H. pylori* infections in childhood anaemia has not been investigated. In low income settings, it may be difficult to exclude other potential causes of childhood anaemia. However, in the Butajira area of southern Ethiopia, where this study cohort is situated, the magnitude of intestinal parasitosis is very low among 3 years old children [27], and malaria transmission is low [28].

We therefore used data from our Ethiopian Birth cohort to assess the association between child's exposure to *H. pylori* infection (measured at age 3, 5, and 6.5 years) and prevalence of anaemia at age 6.5 years. We also tested whether *H. pylori* infection was associated with mean difference in haemoglobin and red cell indices at age 6.5 years. Our group have previously reported *H. pylori* prevalence data at age 3 and age 5 [27, 29]. For the current study, additional data were gathered at age 6.5 years.

## Methods

### Study setting and design

A detailed description of the original Butajira birth cohort study has been published [27, 30]. Briefly, the birth cohort is nested in the Butajira Demographic Surveillance Site (DSS) which covers a sample of nine rural and one urban administrative units in and around the town of Butajira in Southern Ethiopia [31]. Between July 2005 and February 2006, all women in the DSS aged 15–49 and in their third trimester of pregnancy were identified by the DSS fieldworkers and invited to participate in the study. Of the 1,234 eligible women, 1,065 were recruited (86 % of those eligible) and all live singleton babies born to these women (n = 1006) were followed-up as a birth cohort.

### Measurement and data collection

After informed consent forms were signed by the mothers, information on demographic and selected lifestyle factors was collected by interviewer-led administered questionnaire during pregnancy: information on mother’s age, place of residence, ethnicity, religion, occupation, education and household income was collected. At birth and during the follow-up visits, the project data collectors visited the child at home and collected information on potential confounders such as birth weight, history of vaccination, household size, vitamin A supplementation, intestinal parasitosis, anthropometric characteristics and sanitary conditions.

At follow up visits at ages 3, 5 and 6.5 years, mothers were also asked to collect a faecal sample from their child using a leak-proof plastic container. The samples were then transported for analysis in the Butajira health center laboratory to ascertain the child's *H. pylori* and intestinal parasites infection status. Furthermore, at the 6.5 year follow up visit, a blood sample was collected from each child using a vacutainer tube, and transported to Butajira hospital for haematological analysis.

### Laboratory analyses

*H. pylori* status was evaluated using the commercially available SD Bioline H. pylori stool antigen test (Standard Diagnostics, Inc) according to the manufacturer's instructions. A portion of faeces (about 50 mg) from a stool sample was swirled with assay diluent solution at least for ten times, until the sample has been dissolved, and then allowed to settle for 5 min at room temperature. About 100 μL of the prepared sample was placed on the *H. pylori Ag* examination device. The test results were checked about 15 min later. One red line indicated negative and a double red line indicated an *H. pylori* positive result*.*

Additionally, all faecal samples were examined qualitatively using the modified formol-ether concentration method to ascertain the child's intestinal parasites infection status.

### Haematological analysis

At the 6.5-year follow-up, a two ml whole blood sample was collected into Ethylene diaminetetraacetic acid (EDTA) tubes between 8:00 and 10:00 am and analyzed on the same day using an automated haematological analyzer (Cell Dyn 1800, Abbott, USA) at Butajira hospital. The analyzer aspirates the blood sample, dilutes and counts leukocytes, erythrocytes and thrombocytes, measures Mean Cell Volume (MCV) and Haemoglobin (Hb), and calculates Haematocrit, Mean Cell Haemoglobin (MCH), and Mean Cell Haemoglobin Concentration (MCHC). This instrument was monitored daily with normal, high and low controls provided by the manufacturer before running the specimen to ensure quality of haematological analyses.

### Outcome definition

The primary study outcome was anaemia at age 6.5, and was defined according to the WHO hemoglobin cutoff: < 11.5 g/dL for children 5–11 years [32].

### Statistical analysis

Data were double-entered into EpiData 3.1 (EpiData, Denmark). The datasets were cleaned, coded and merged ready for analysis using Stata 12 (Statacorp, College Station, Texas, USA).

Prior to investigating the association between *H. pylori* infection and anaemia, univariate analyses were used to identify the possible confounders. Variables that were associated with both exposure and outcome variables in the crude analysis using statistical significance at p value <0.2 were considered to be possible confounders. These included place of residence, ethnicity, religion, maternal education, source of water, crowdedness, sanitary conditions, history of vaccination and history of vitamin A supplementation. Additionally, we included variables previously shown to be associated with anaemia in the literature; child’s sex, anthropometric measures of nutritional status and intestinal parasite status [33].

The hypothesis that infection with *H. pylori* would be associated with anaemia (a categorical variable) was assessed using univariate and multivariate logistic regression models. To evaluate the effect of *H. pylori* on haemoglobin and red cell indices as continuous outcomes (*Y*), regression analyses were performed using generalized linear models (GLIM) with random response variables, Hb, RBC, MCV, MCH and MCHC assuming a gamma distribution for *Y*. Maximum likelihood allowed estimation of column vector of coefficients, β, in the function *f*(μ*Y*) = Xβ, where *f*( ) is the link function. We chose the logarithm link (log(*μY*) = Xβ), based on the optimization of both the Akaike Information Criterion and the Bayesian Information Criterion [34].

We first examined the crude association between child's *H. pylori* infection (measured at age 3, 5, 6.5 years) and outcome variables (anaemia, haemoglobin and red cell indices) at age 6.5 years. We then repeated the analysis, adjusting for the possible confounders and predictors of outcome listed in Additional file 1: Table S1.

The same approach was used in a separate set of analyses to assess the association between *H. pylori* infection and anaemia, haemoglobin level and red cell indices for all available children at year 6.5, by creating a new exposure variable with categories representing different combinations of *H. pylori* exposure status at age 3, 5 and 6.5: ‘never infected’ (never infected at any of these three time points) and 'infected any age up to year 6.5’. Covariates were kept in the model if they changed the coefficient of exposure (*H. pylori* infection) by > 10 % or if they were independently associated with the outcome at *p* < 0.10. Probability values < 0.05 were considered statistically significant for main effects. Sensitivity analysis was done to compare the distribution of demographic and life style variables between study subject who have complete outcome data (i.e. “complete-case”) and "all respondents" populations.

### Ethical approval

The study was approved by the Institutional Review Board (IRB) of Addis Ababa University, College of Health Sciences, Ethiopia. Written, informed consent was obtained from the mothers after they have been clearly informed about the study, and in keeping with the requirements of the College of Health Sciences IRB all women and their children were reimbursed for health care costs. Children were also requested to give assent and were informed of their right to refuse to participate in the study and to withdraw at any time during the study without jeopardizing their right of access to other health services. Invasive procedures such as collection of blood samples were fully explained to parents and children, and were carried out using sterile disposable materials.

## Results

### Description of cohort participants followed-up at age 6.5 years

**Fig. 1:**
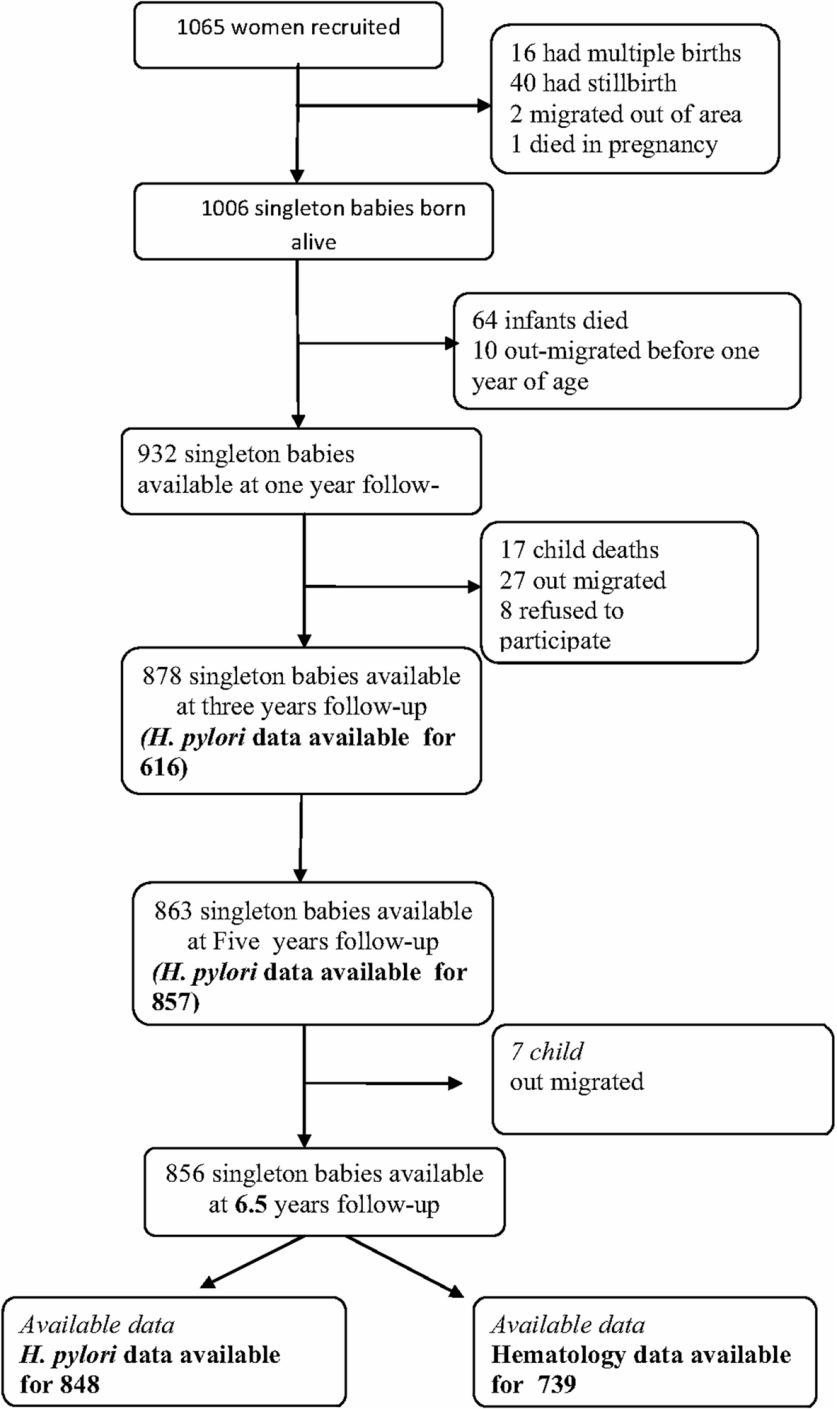
The study cohort

At recruitment, a total of 1,006 singleton babies made up the initial birth cohort, of which 64 (6.4 %) had died and 10 (0.9 %) had migrated from the study area before their first birthday. A detailed description of the cohort at years 1, 3, and 5 is reported elsewhere [27, 29, 31]. At 6.5 years, a total of 856 singleton children were successfully followed-up (85.1 % of the original cohort at birth, and 99.3 % of those available at year 5 follow-up), of whom 739 had haematology and 848 had *H. pylori* data at the 6.5 year follow up visit (Fig. 1). Comparing the distribution of demographic and life style variables between study subjects who have complete outcome data (i.e. “complete-case” and "all respondents"), found similar patterns of distribution in relation to demographic and life style characteristics (Additional file 2: Table S3).

### Selected demographic and lifestyle characteristics of children and their mothers at the 6.5 year follow-up visit

Of children enrolled at the 6.5 year follow up visit, 51.2 % (434/848) were male and the majority, 88.2 % (748/848) were from a rural area. Maternal demographic characteristics showed that 47.4 % (402/848) of the mothers belonged to the Meskan ethnic group, 78.4 % (665/848) were Muslim, 71.6 % (607/848) were illiterate and 83.8 % were housewives (709/848). Most mothers (61.3 %, 520/848) reported using piped water as their primary drinking source. Selected early life characteristics at 2 months and 1 year of age showed that 57.5 % had been vaccinated at 2 months and only 11.7 (99/848) had received vitamin A supplementation at 1 year. Only 60 % of children had birth weight data, and of these, 91.3 % (483/529) were of normal birth weight (Table 1).

**Table 1:** Selected demographic and lifestyle characteristics of children and their mothers at 6.5 year follow-up visit, Butajira Birth Cohort, Ethiopia

Variables	Number	Percent
**Sex**		
Male	434	51.2
Female	414	48.8
**Place of residence**		
Urban	100	11.8
Rural	748	88.2
**Ethnicity**		
Meskan	402	47.4
Mareko	111	13.1
Silti	200	23.6
Others	135	15.9
**Religion**		
Orthodox Christian	121	14.3
Muslim	665	78.4
Catholic	10	1.2
Protestant	52	6.1
Maternal age Mean (SD)	27.1 (6.27)	
**Maternal age group**		
15-24	316	37.3
25-34	134	41.7
35-44	133	15.7
**Maternal education**		
None	607	71.6
Informal only	83	9.8
Formal	158	18.6
**Maternal occupation**		
Housewife	709	83.8
Farming and related	27	3.2
Trading and related	96	11.3
Other	14	1.7
**History of vaccination at 2 months**		
Vaccinated	485	57.5
Not vaccinated	359	42.5
Birth weight (N = 529)		
Normal	483	91.3
Low (<2.5 kg)	46	8.7
**History of any vaccination at 1 year**		
Had some vaccination	823	97.6
Not vaccinated at all	20	2.4
**Sanitary conditions**		
Poor	156	18.4
Good	692	81.6
**Water source**		
River	179	21.2
Well	134	15.8
Pipe	520	61.5
Spring	13	1.5
**Vitamin A supplementation at 12 months**		
Yes	99	11.7
No	747	88.3

### Anaemia and haematology parameters among children followed up at age 6.5

The prevalence of anaemia among children enrolled at the 6.5 year follow up visit was 34.8 % (257/739), and the mean (SD) hematological parameters were: haemoglobin concentration 11.8 (1.1) gm/dl; red blood cells 4.5 (0.37) × 10^12^/L; mean cell haemoglobin 26.1 (2.2) pg; mean cell haemoglobin concentration 31.6 (1.5) gm/dl; mean cell volume 81.1 (4.8) fl. No significant differences in haematological parameters between males and females were observed. Likewise, the prevalence of anaemia did not differ significantly between sexes, (33.0 % vs 36.5 % in female and males, respectively, p = 0.31, Table 2).

**Table 2:** Haematological parameters mean (standard deviation) and anaemia prevalence at age 06.5 years according to gender of child, Butajira birth cohort study, Ethiopia. N = 739

Variables	Overall (N = 739)	Female N = 361	Male (N = 378)	Mean difference/Crud OR (95 % CI)	p value
Hgb (gm/dl)	11.8 (1.1)	11.8 (1.08)	11.7 (1.15)	0.12 (−0.04-0.28)^a^	0.14
RBC (x10^12^/L)	4.5 (0.37)	4.51 (0.35)	4.52 (0.37)	0.12 (−0.04-0.06)^a^	0.66
MCH (pg)	26.1 (2.2)	26.2 (2.16)	26.1 (2.24)	0.13 (−0.19-0.45)^a^	0.43
MCHC (gm/dl)	31.6 (1.5)	31.8 (1.35)	31.7 (1.56)	0.02 (−0.18-0.23)^a^	0.82
MCV (fl)	81.1 (4.8)	81.3 (4.69)	81.0 (4.82)	0.40 (−0.28-1.09)^a^	0.25
Anaemia prevalence^¥^ n(%)	257 (34.8)	119 (33.0)	138 (36.5)	0.85 (0.63-1.15)^b^	0.31

^¥^Anaemia was defined according to WHO haemoglobin cut-offs: < 11.5 g/dL for children 5–11 years

^a^Mean difference and 95 % CI mean difference was calculated using the independent t test

^b^Crude Odds ratio (OR) and 95 % CI OR was calculated using binary logistic regression

### Prevalence of *H. pylori* infection at age 3, 5 and 6.5 years

*H. pylori* infection was reported in 41 % of children at age three [27] and 44 % at age five [29] respectively. However a sharp declining of *H. pylori* infection 10.4 % (88/848) was found at age 6.5 years. The prevalence of *H. pylori* infection at age 6.5 was significantly differ by urban or rural area of residence such that 17 % (17/100) of urban children and 9.5 % (71/748) of rural children have current infection (p < 0.01) (Additional file 1: Table S1).

### Relation between potential confounders with anaemia and *H. pylori* infection

Effects of potential confounders on outcomes and exposures showed no statistically significant association with most demographic and lifestyle variables including; child's sex, religion, history of vaccination, birth weight, history of vitamin A supplementation at 1 year, history of vaccination at 2 months, sanitary conditions, crowdedness or intestinal parasite infection at age 6.5. However, both *H. pylori* exposure at age 6.5 and *H. pylori* exposure at any age up to 6.5 were significantly associated with place of residence, while, anaemia was significantly associated with place of residence, source of water, maternal education and ethnic group (Additional file 1: Table S1).

### Association between *H. pylori* infection and Anaemia at age 6.5

At the 6.5 year follow up visit, univariate analysis showed that the prevalence of anemia was higher in children infected with *H. pylori* (37.0 %) than in those not infected (34.5 %), but the difference was not significant (p = 0.38). Children who had exposure to *H. pylori* infection at ages 3 and 5 were found to have higher prevalence of anaemia at age 6.5, and the latter association reached statistical significance (p < 0.05). Furthermore, any *H. pylori* infection between 3 and 6.5 years of age was associated with a higher prevalence of anaemia at age 6.5 years (Additional file 3: Table S2).

In multivariate analysis adjusted for *a priori* confounders, *H. pylori* infection at ages 3 and 6.5 was positively, though not significantly, related to prevalence of anaemia at age 6.5 years (adjusted OR, 95 % CI, 1.16; 0.79 to 1.71, p = 0.44 and 1.15; 0.69 to 1.93, p = 0.59 respectively). There was an increased odds of anaemia at age 6.5 years given *H. pylori* infection at age 5 (adjusted OR, 95 % CI, 1.52; 1.11 to 2.08, p = 0.01, Table 3). Furthermore, separate analysis considering any *H. pylori* infection up to age 6.5 showed that increased odds of anaemia among children ever infected with *H. pylori* (adjusted OR, 95 % CI, 1.68; 1.22 to 2.32, p = 0.01, Table 3).

**Table 3:** Associations between anaemia at age 6.5 years and *H. pylori* infection at the age of three, 5, and 6.5 years, Butajira birth cohort study, Ethiopia

	Anaemia at age 6.5 years^a^						
Variables	N	Yes N (%)	No N (%)	Crude OR (95 % CI)	P-value	Adjusted OR^c^ (95 % CI)	P-value
**Exposure to** ***H. pylori*** **at age 3**							
No	300	102 (34.0)	198 (66.0)	1		1	
Yes	212	77 (36.3)	135 (67.7)	1.12 (0.76-1.60)	0.58	1.16 (0.79-1.71)	0.44
**Exposure to** ***H. pylori*** **at age 5**							
No	406	124 (30.5)	282 (69.5)	1		1	
Yes	323	128 (39.6)	195 (60.4)	1.49 (1.09-2.02)	0.01	1.52 (1.11-2.08)	0.01
**Exposure to** ***H. pylori*** **at age 6.5**							
No	666	230 (34.5)	436 (65.5)	1		1	
Yes	73	27 (37.0)	46 (63.0)	1.11 (0.67-1.83)	0.67	1.15 (0.69-1.93)	0.59
**Exposure to** ***H. pylori*** **up to age 6.5** ^**b**^							
Never infected	309	88 (28.5)	221 (71.5)	1		1	
Infected at any age up to age 6.5	430	169 (39.3)	261 (60.7)	1.62 (1.19-2.23)	0.01	1.68 (1.22-2.32)	0.01

^a^Aanemia was defined according to WHO haemoglobin cutoffs: < 11.5 g/dL for children 5–11 years

^b^H pylori infection at any age from 3 to 6.5 years

^c^Adjusted for, child’s gender, area of residence, maternal education, history of vaccination at 2 months, vitamin A supplementation at 1 year, sanitary condition, source of water and child’s height at age 6.5

### Association between *H. pylori* infection and haemoglobin level at age 6.5

Linear regression models related haemoglobin concentration (outcomes) to the individual estimates of *H. pylori* infection status at ages 3, 5 and 6.5 years (exposures). These showed a non-significant reduction in haemoglobin concentration for children infected with *H. pylori* at age 6.5 compared to non infected children (adjusted β = −0.08; 95 % CI, −0.34 to 0.17, p = 0.52), whilst the same analysis for children infected at age 5 showed a statistically significant reduction in haemoglobin concentration (adjusted β = −0.20, 95 % CI; −0.37 to −0.04, p < 0.01). When haemoglobin concentrations among all children followed up to age 6.5 years were regressed against any *H. pylori* infection up to age 6.5, being infected was associated with a significantly lower haemoglobin concentration (adjusted β = −0.19, 95 % CI, −0.35 to −0.03, p = 0.01, Table 4).

**Table 4:** Multivariate generalized linear model of haematological parameters at age 6.5 years in association with *Helicobacter pylori* infection from 3 to 6.5 years of age, Butajira birth cohort study, Ethiopia

	Hb			RBC			MCV			MCH			MCHC		
Variables	β^a^	95 % CI	P	β^a^	95 % CI	P	β^a^	95 % CI	P	β^a^	95 % CI	P	β^a^	95 % CI	P
**Exposure to** ***H. pylori*** **at age 3**															
Yes	0.07	0.27 to −0.15	0.49	0.02	−0.04 to 0.08	0.45	−0.47	−1.36 to 0.43	0.30	−0.04	−0.45 to 0.37	0.85	−0.13	−0.40 to 0.14	0.33
No	0^b^			0^b^			0^b^			0^b^			0^b^		
**Exposure to** ***H. pylori*** **at age 5**															
Yes	−0.20	−0.37 to −0.04	0.01	−0.02	−0.07 to −0.03	0.44	−0.25	−0.97 to 0.46	0.48	−0.26	−0.59 to 0.07	0.12	−0.12	−0.33 to 0.10	0.29
No	0^b^			0^b^			0^b^			0^b^			0^b^		
**Exposure to** ***H. pylori*** **at age 6.5**															
Yes	−0.08	−0.34 to 0.17	0.52	−0.03	−0.12 to 0.56	0.49	−0.04	−1.22 to 1.14	0.94	0.23	−0.32 to 0.78	0.41	−0.06	−0.42 to 0.29	0.73
No	0^b^			0^b^			0^b^			0^b^			0^b^		
**Exposure to** ***H. pylori*** **up to age 6.5**															
Infected at any age up to age 6.5	−0.19	−0.35 to −0.03	0.01	−0.06	−0.15 to 0.02	0.11	−2.22	−3.43 to −1.01	0.01	−0.63	−1.15 to - 0.12	0.01	−0.67	−1.21 to −0.14	0.01
Never infected	0^b^			0^b^			0^b^			0^b^			0^b^		

^a^Changes for haematological parameters at age 6.5 years by *Helicobacter pylori* infection status from 3 to 6.5 years of age, adjusted for child’s gender, area of residence, maternal education, history of vaccination at 2 months, source of water, child’s height and weight at 6.5 years

^b^Reference group

### Association between *H. pylori* infection and red cell indices at age 6.5

Separate linear regression models related red cell indices (outcomes) to the individual estimates of *H. pylori* infection status at ages 3, 5 and 6.5 years (exposures). These showed a non-significant reduction for most red cell indices measured at age 6.5 years among *H. pylori* infected children compared to non-infected children. When red cell indices among all children followed up to age 6.5 years were regressed against any *H. pylori* infection up to age 6.5 years, being infected was associated with lower red cell indices; MCV (adjusted β = −2.22, 95 % CI, −3.43 to −1.01, p = 0.01), MCH (adjusted β = −0.63, 95 % CI, −1.15 to −0.12, p = 0.01) and MCHC (adjusted β = −0.67, 95 % CI, −1.21 to −0.14, p = 0.01) respectively (Table 4).

## Discussion

In this population-based birth cohort of young Ethiopian children followed-up to age 6.5 years, we have examined the association between earlier and current *H. pylori* infection and anaemia at age 6.5 years. We found that current *H. pylori* infection at age 6.5 years was positively, though not significantly, related to prevalence of anaemia, while any *H. pylori* infection up to age 6.5 years was significantly associated with an increased odds of anaemia.

The strengths of this study are that the data come from a population based birth cohort with a high response rate and good retention (93 % of surviving mother-child pairs were retained between birth and 6.5 years), thereby minimizing selection bias. We have also used a highly sensitive and specific *H. pylori* stool antigen test [35]. In addition, measurement of the key outcome (anaemia) was done objectively using WHO age-specific haemoglobin cutoffs [32], though identification of anaemia by means of the haemoglobin level may have impaired the detection of iron-deficiency anemia, which might be better detected by measuring serum ferritin and transferrin receptor [36]. This methodological approach however still requires validation in population surveys [37] and in resource limited settings, the prevalence of iron deficiency has often been derived from the prevalence of anaemia using measurements of blood haemoglobin [38–40], and red cell indices [41]. Our data showed a significant reduction of Hb, MCV and MCH among children who had any *H. pylori* infection up to age 6.5*,* which provide, in part, an indication that iron-deficiency as a plausible origin for the observed anemia. Although other iron-related tests are required for the confirmation of iron deficiency, it is reasonable to assume that a population with a high anemia prevalence is likely to also have a high prevalence of iron deficiency [38, 40]. Furthermore, hereditary hemoglobinopathies are uncommon in Ethiopia [42], so bias relating to either missing or misclassifying the other forms of anaemia in our study population is unlikely to be a serious concern.

The decrease prevalence of *H. pylori* infection in our study population (10.4 %) of six and half year old children, can be contrasted with the previous reports of 41 % at age three [27] and 44 % at age five [29] in the same cohort. However, a similar pattern of *H. pylori* prevalence was reported in previous cohort study in Butajira, where the peak age for *H. Pylori* infection was reported below 6 year, and patterns of repeated seroconversion and sero-reversion are restricted in early childhood [24]. Another cohort study among children from high income country also reported a decreased prevalence of *H. pylori* infection in older children from 13.6 % at age 18 and 24 months to only 3 % at age 11 years [43]. The authors suggested that infection with *H. pylori* occurred at an early age and spontaneously cleared later [43].

The decline in prevalence of *H. pylori* at age 6.5 in this study may be due to spontaneous elimination of the bacterium [43], or better attention to health issues in older children, or use of antibiotics for other common diseases [44]. Another explanation of this finding could be an increasing antibody production with increasing age that may lead to the decline of the prevalence rate in older children [45].

The results of this study should be interpreted with caution because the study was conducted in Ethiopia, a low income country in which the causes of anaemia are multi-factorial. Demographic variables and markers of socio-economic status have commonly been found to be associated with anaemia [33, 46, 47]. We therefore measured and controlled for markers of socio-economic status, demographic and lifestyle variables, and found no evidence of confounding by markers of socioeconomic status (urban or rural residence, maternal education, maternal occupation, sanitary condition, source of water or crowdedness), demographic or lifestyle variables (sex, birth weight, history of vaccination at 2 months and 1 year, or vitamin A supplementation at 1 year), which suggest that the effects seen are unlikely to be caused by residual confounding by markers of socio-economic status, demographic and lifestyle variables.

Parasitic diseases causing anaemia are common in Ethiopia, with a recent survey reporting 7.9 % to 34.6 % of school-aged children infected with soil-transmitted helminths, 2.9 % with schistosomiasis and 0.6 % with malaria, respectively [48, 49]. However, we found no evidence that intestinal parasite infections were contributing to the anaemia we observed at age 6.5.

Infection with malaria parasites has also been associated with anaemia in Ethiopia [33, 47], but our analysis lacked data on status of malaria infection. Studies in the Butajira area of southern Ethiopia have previously shown that malaria transmission is very low. A large population based longitudinal study documented a very low prevalence of malaria 0.93 % (178 malaria cases among 19,207 people) during the peak season for malaria transmission in the area [28]. Another large longitudinal study also documented a very low prevalence of malaria (1.5 % (16/1080)), with 0 % during December [50]. This suggests that malaria is unlikely to be a source of bias. However, the observed association between *H. pylori* infection and anemia in this study might not be the same in areas where malaria transmission is high. Further study should be done at high malaria burden settings.

Poor nutrition can cause iron deficiency and potentially increase susceptibility to *H. pylori* infection. However, the consistent effect estimates observed for the different outcomes (anaemia, haemoglobin level and red cell indices) suggest that the findings did not result from residual confounding by malnutrition, or failure to adjust for other potential confounders.

The significant association between any *H. pylori* infection at any age up to 6.5 years and anaemia in the current study is backed by previous epidemiological studies in different settings [51, 52]. A study among Arab children (age 6–9) in Israel reported a 2.8-fold higher risk of anaemia in *H. pylori* infected children compared with uninfected children, after controlling for socioeconomic confounders [51]. Our findings are also consistent with data reported by Baggett *et al.* [52] from rural Alaska who found a 4.1-fold higher risk of anaemia (AOR: 4.1; 95 % CI, 0.92 to 8.0; *p = 0*.06) in children (mean age 9.5 years) infected with *H. pylori*. Another study in the United States by Cardenas *et al*. [53] used data from the 1999–2000 *National Health and Nutrition Examination Survey* (NHANES), with 7,462 individuals aged ≥3 years. This reported a significant association between sero-positivity for *H. pylori* and iron deficiency anaemia (OR = 2.6; 95 % CI: 1.5 to 4.6) and other types of anaemia (OR = 1.3; 95 % CI: 1.0 to 1.7). The difference in size of the ORs may be due to variation in age, outcome ascertainment, and differences in measurement of infection status.

In this study, the exposure variable representing current *H. pylori* infection (positive for stool antigen test) at age 6.5 was not significantly related to prevalence of anemia. Other studies have also shown no associations between acute/current *H. pylori* infection and childhood anaemia [54, 55]. The lack of association between acute *H. pylori* infection and anaemia has been speculated to relate to different stages or expressions of *H. pylori* infection [54]. For instance, current/acute *H. pylori* infection assessed with stool antigen test [35] may reflect initial *H. pylori* acquisition, before the establishment of the bacteria, the development of the subsequent immune response, and any effects on the child’s haematopoetic system. The bacteria may exist transiently for some time in the stomach of children before persistent colonization and becoming an established infection affecting iron metabolism and causing anemia [54]. It is possible that children positive for stool antigen at age 6.5 years in the study may have been exposed to *H. pylori* for only a short time, so iron stores and haemoglobin synthesis are not yet affected. In contrast to this hypothesis, however, Queiroz *et al.* [56] suggested that initial colonisation of *H. pylori* could provoke a stronger inflammatory response, which in turn inhibits iron absorption. This earlier study, unlike the current one, was done among symptomatic children who underwent upper gastrointestinal endoscopy, and may account for higher inflammatory responses. Our study population was distinctly different as all our participants were apparently healthy children, and the former hypothesis seems more likely to fit with our observations.

We have also explored the association between *H. pylori* infection and haemoglobin levels and red cell indices at age 6.5 years and found a significant reduction of haemoglobin concentration (adjusted β = −0.19gm/dl, 95 % CI, −0.35 to −0.03, p = 0.01) among children infected to *H. pylori* at any age up to 6.5 years. This finding also fit with previous studies by both Muhsen *et al.* [51] *and* Queiroz *et al.* [57]*,* who report a significant reduction in haemoglobin level among *H. pylori* infected children, respectively. Similar to the trend seen for haemoglobin, significant reductions in MCV, MCH and MCHC were observed in *H. pylori* infected subjects, which may indicate anaemia from iron deficiency. A large household controlled therapeutic trial among children with iron deficiency aged 7–11 years showed that anti-*H. pylori* therapy reduced the prevalence of iron deficiency anaemia substantially, and reduced iron deficiency more modestly 40 months after treatment initiation, suggesting that *H. pylori* may play a causal role in haematological outcomes [58].

Several logical mechanisms of association between anaemia and *H. pylori* infection have been suggested. Studies have shown that the *H. pylori* colonization is associated with reduction in iron absorption due to low levels of gastric acid [21], effects on iron transporter molecules [22], blood loss due to *H. pylori*-induced gastritis or duodenitis [59] and sequestration of dietary iron by *H. pylori* residing in the gastric mucosa [60]. Increased hepcidin production from hepatocytes in response to interleukin-6 ( IL-6) production in *H. pylori* gastritis has also been proposed as possible mechanism to explain *H. pylori* associated IDA [23], as this prevents the release of iron from stores in enterocytes.

## Conclusions

In conclusion, our findings indicate that any previous *H. pylori* infection is associated with higher prevalence of anaemia and reduction of haemoglobin level and red cell indices in school-age children, independent of socioeconomic variables. However, current *H. pylori* infection was not significantly associated with increased prevalence of anaemia. Further investigation of the natural history of *H. pylori* infection will be crucial to understanding its manifestations in young children from developing countries, and to develop treatment guideline for *H. pylori* infected children with unexplained anaemia.

## Additional files

Additional file 1: Table S1. Correlates of *Helicobacter pylori* infection and Anaemia among children aged 6.5 years, Butajira Birth Cohort, Ethiopia. Additional file 2: Table S3. Comparison of the distribution of demographic and lifestyle characteristics in “Case-complete” and “All respondents” childern at 6.5 year follow-up visit, Butajira Birth Cohort, Ethiopia. Additional file 3: Table S2. Haematological Parameters Mean (Standard deviation) and Anaemia Prevalence at Age 6.5 Years According to Child's *H. pylori* infection status from 3 to 6.5 Years of Age, Butajira Birth Cohort Study, Ethiopia.
