# Supplementary material for: Effect of early and current Helicobacter pylori infection on the risk of anaemia in 6.5-year-old Ethiopian children
Source: BMC Infect Dis. 2015 Jul 14;15:270. doi: 10.1186/s12879-015-1012-y (PMC4501201; doi:10.1186/s12879-015-1012-y)
Supplement: Additional file 1: Table S1. — Correlates of Helicobacter pylori infection and Anaemia among children aged 6.5 years, Butajira Birth Cohort, Ethiopia. [file 12879_2015_1012_MOESM1_ESM.doc]

**Table S1.** Correlates of *Helicobacter pylori* infection and Anaemia among children aged 6.5 years, Butajira Birth Cohort, Ethiopia.

| **Variables** | **Overall**  **N (%)** | **Exposure to *H pylori* at age 6.5 (N=848)** | **Exposure to *H pylori* up to 6.5**^¥^ **(N=848)** | **Overall**  **N (%)** | **Anaemia**^¥¥^  **(N=739)** | **Crude OR**  **(95% CI)** ^¥¥¥^ | **Crude OR**  **(95% CI)** ^¥¥¥¥^ | **Crude OR**  **(95% CI)** ^¥¥¥¥¥^ |
| --- | --- | --- | --- | --- | --- | --- | --- | --- |
|  |  | **Yes**  **N (%)** | **Yes**  **N (%)** |  | **Yes**  **N (%)** |  |  |  |
| **Sex** |  |  |  |  |  |  |  |  |
| Female | 414 (48.8) | 46 (11.1) | 242 (58.5) | 361 (48.9) | 119 (33.0) | 1.16 (0.75-1.81) | 1.02 (0.78-1.34) | 0.86 (0.63-1.15) |
| Male | 434 (51.2) | 42 (9.7) | 251 (57.8) | 378 (51.1) | 138 (36.5) | 1 | 1 | 1 |
| **Place of residence** |  |  |  |  |  |  |  |  |
| Rural | 748 (88.2) | 71 (9.5) | 423 (56.6) | 653 (88.4) | 242 (37.1) | 0.51 (0.28-0.91)* | 0.56 (0.35-0.87)* | 2.78 (1.56-4.97)* |
| Urban | 100 (11.8) | 17 (17.0) | 70 (70.0) | 86 (11.6) | 15 (17.4) | 1 | 1 | 1 |
| **Ethnicity** |  |  |  |  |  |  |  |  |
| Meskan | 402 (47.4) | 48 (11.9) | 233 (58.0) | 339 (45.9) | 100 (29.5) | 1.52 (0.76-3.03)* | 0.91 (0.61-1.36) | 1.06 (0.67-1.67) |
| Mareko | 111 (13.1) | 9 (8.10) | 61 (55.0) | 103 (13.9) | 48 (46.4) | 0.99 (0.39-2.49) | 0.81 (0.48-1.35) | 2.21 (1.28-3.84)* |
| Silti | 200 (23.6) | 20 (10.0) | 118 (59.0) | 173 (23.4) | 74 (42.8) | 1.25 (0.58-2.70) | 0.95 (0.61-1.49) | 1.90 (1.16-3.11)* |
| Others | 135 (15.9) | 11 (8.10) | 81 (60.0) | 124 (27.4) | 35 (16.8) | 1 |  | 1 |
| **Religion** |  |  |  |  |  |  |  |  |
| Muslim | 665 (78.4) | 74 (11.1) | 382 (57.4) | 571 (73.3) | 204 (35.7) | 1.51 (0.83-2.74)* | 0.87 (0.62-1.22) | 1.20 (0.83-1.74) |
| Christian | 183 (21.6) | 14 (7.7) | 111 (60.7) | 168 (22.7) | 53 (31.5) | 1 | 1 | 1 |
| **Maternal education** |  |  |  |  |  |  |  |  |
| None | 607 (71.6) | 62 (10.2) | 355 (58.5) | 535 (72.4) | 195 (36.4) | 0.78 (0.45-1.34) | 1.12 (0.78-1.59) | 1.62 (1.06-2.47)* |
| Informal only | 83 (9.8) | 6 (7.2) | 50 (60.2) | 70 (9.5) | 27 (38.6) | 0.53 (0.20-1.39) | 1.20 (0.70-2.06) | 1.77 (0.95-3.29)* |
| Formal | 158 (18.6) | 20 (12.7) | 88 (55.7) | 134 (18.1) | 35 (26.1) | 1 | 1 | 1 |
| **Maternal occupation** |  |  |  |  |  |  |  |  |
| Housewife | 709 (83.6) | 70 (9.9) | 408 (57.5) | 615 (83.2) | 219 (35.6) | 0.43 (0.12-1.59) | 0.49 (0.15-1.56) | 0.99 (0.33-3.00) |
| Farming and related | 28 (3.3) | 3 (10.7) | 16 (57.1) | 26 (3.5) | 7 (26.9) | 0.48 (0.08-2.74) | 0.48 (0.12-1.90) | 0.66 (0.16-2.67) |
| Trading and related | 96 (11.3) | 12 (12.5) | 58 (60.4) | 84 (11.4) | 26 (31.0) | 0.57 (0.14-2.32) | 0.55 (0.16-1.87) | 0.80 (0.24-2.64) |
| Other | 15 (1.8) | 3 (20.0) | 11 (73.3) | 14 (1.9) | 5 (35.7) | 1 | 1 | 1 |
| **Maternal age** |  |  |  |  |  |  |  |  |
| 15-24 | 316 (37.3) | 37 (11.7) | 175 (55.4) | 257 (37.2) | 87 (31.6) | 1 | 1 | 1 |
| 25-34 | 399 (47.1) | 37 (9.3) | 236 (59.1) | 350 (47.4) | 123 (35.1) | 0.77 (0.47-1.24) | 1.16 (0.86-1.57) | 1.09 (0.78-1.53) |
| 35-44 | 133 (15.7) | 14 (10.5) | 82 (61.7) | 114 (15.4) | 47 (41.2) | 0.88 (0.46-1.70) | 1.29 (0.85-1.96) | 1.22 (0.77-1.92) |
| **Water source** |  |  |  |  |  |  |  |  |
| River or Spring | 192 (22.7) | 17 (8.9) | 103 (53.6) | 166 (22.5) | 45 (27.1) | 0.75 (0.42-1.31) | 0.74 (0.53-1.04)* | 0.62 (0.42-0.92)* |
| Well | 134 (15.8) | 11 (8.2) | 72 (53.7) | 118 (16.0) | 42 (35.6) | 0.68 (0.35-1.34) | 0.75 (0.51-1.09) | 0.93 (0.61-1.42) |
| Pipe | 520 (61.3) | 60 (11.5) | 316 (60.8) | 453 (61.5) | 169 (37.3) | 1 | 1 | 1 |
| **History of vaccination^∞^** |  |  |  |  |  |  |  |  |
| Not vaccinated | 359 (42.5) | 29 (8.10) | 197 (54.9) | 307 (41.7) | 105 (34.2) | 0.64 (0.40-1.03) | 1.25 (0.95-1.65)* | 0.97 (0.71-1.33) |
| Vaccinated | 485 (57.5) | 58 (12.0) | 293 (60.4) | 429 (58.5) | 149 (34.7) | 1 | 1 | 1 |
| **Birth weight (N=529)** |  |  |  |  |  |  |  |  |
| Low (<2.5Kg) | 46 (8.7) | 3 (6.5) | 26 (56.5) | 39 (8.2) | 17 (43.6) | 0.56 (0.17-1.88) | 1.04 (0.56-1.91) | 1.14 (0.58-2.21) |
| Normal | 483 (91.3) | 53 (11.0) | 268 (55.5) | 436 (91.8) | 176 (40.3) | 1 | 1 | 1 |
| **Vitamin A supplementation^∞∞^** |  |  |  |  |  |  |  |  |
| No | 743 (88.2) | 78 (10.5) | 434 (58.4) | 644 (87.7) | 228 (35.4) | 1.04 (0.52-2.09) | 0.79 (0.60-1.05) | 1.37 (0.84-2.24)* |
| Yes | 99 (11.8) | 10 (10.1) | 57 (57.6) | 90 (12.3) | 26 (28.9.2) | 1 | 1 | 1 |
| **Poor sanitary conditions*^∞∞∞^*** |  |  |  |  |  |  |  |  |
| No | 692 (81.6) | 75 (10.8) | 404 (58.4) | 609 (82.4) | 218 (35.8) | 0.75 (0.41-1.38) | 0.95 (0.67-1.35) | 0.77 (0.51-1.15)* |
| Yes | 156 (18.4) | 13 (8.3) | 89 (57.1) | 130 (17.6) | 39 (30.0) | 1 | 1 | 1 |
| **Crowdedness*^∞∞∞∞^*** |  |  |  |  |  |  |  |  |
| 1-2 | 469 (55.3) | 46 (9.8) | 268 (57.1) | 339 (54.0) | 139 (34.8) | 1 |  | 1 |
| 3-4 | 298 (35.1) | 36 (12.1) | 184 (61.7) | 266 (36.0) | 96 (36.1) | 1.26 (0.79-2.00) | 1.21 (0.90-1.62)* | 1.05 (0.76-1.46) |
| 5-12 | 81 (9.6) | 6 (7.4) | 41 (50.6) | 74 (10.0) | 22 (29.7) | 0.73 (0.30-1.78) | 0.76 (0.47-1.23) | 0.79 (0.46-1.35) |
| **Intestinal parasitosis** |  |  |  |  |  |  |  |  |
| **Yes** | 218 (26.5) | 22 (10.1) | 124 (56.9) | 200 (27.1) | 69 (34.5) | 0.94 (0.56-1.57) | 0.91 (0.67-1.25) | 0.98 (0.69-1.38) |
| **No** | 604 (73.5) | 64 (10.6) | 356 (58.9) | 539 (71.9) | 188 (34.9) | 1 |  | 1 |

¥ *H pylori infection at any age from 3-6.5 years, ∞ measured at 2month, ∞∞ measured at year 1, ∞∞∞ poor sanitary condition was defined as not having a toilet facility and not having safe water,  ^∞∞∞∞ measured in Person/Household^*

¥¥Anaemia was defined according to WHO haemoglobin cutoffs: < 11.5 g/dL for children 5-11 years

¥¥¥ OR (95% CI) calculated for *H pylori* *infection*  at age 6.5 and covariates

¥¥¥¥ OR (95% CI) calculated for *H pylori* *infection* *at any age from 3-6.5 years* and covariates

¥¥¥¥¥ OR (95% CI) calculated for Anaemia *at age 6.5 years* and covariates

*P value <0.2
