# Supplementary material for: Effect of early and current Helicobacter pylori infection on the risk of anaemia in 6.5-year-old Ethiopian children
Source: BMC Infect Dis. 2015 Jul 14;15:270. doi: 10.1186/s12879-015-1012-y (PMC4501201; doi:10.1186/s12879-015-1012-y)
Supplement: Additional file 2: Table S3. — Comparison of the distribution of demographic and lifestyle characteristics in “Case-complete” and “All respondents” childern at 6.5 year follow-up visit, Butajira Birth Cohort, Ethiopia. [file 12879_2015_1012_MOESM2_ESM.doc]

**Table S3. Comparison of the distribution of demographic and lifestyle characteristics in “Case-complete” and “All respondents” children at 6.5 year follow-up visit, Butajira Birth Cohort study, Ethiopia**

| **Variables** | **All respondent (N=848)**  **N (%)** | **Case complete (N=739)**  **N (%)** |
| --- | --- | --- |
| **Sex** |  |  |
| Female | 414 (48.8) | 361 (48.8) |
| Male | 434 (51.2) | 378 (51.2) |
| **Place of residence** |  |  |
| Rural | 748 (88.2) | 653 (88.4) |
| Urban | 100 (11.8) | 86 (11.6) |
| **Ethnicity** |  |  |
| Meskan | 402 (47.4) | 339 (45.9) |
| Mareko | 111 (13.1) | 103 (13.9) |
| Silti | 200 (23.6) | 173 (23.4) |
| Others | 135 (15.9) | 124 (16.8) |
| **Religion** |  |  |
| Muslim | 665 (78.4) | 571 (77.3) |
| Christian | 183 (21.6) | 168 (22.7) |
| **Maternal education** |  |  |
| None | 607 (71.6) | 535 (72.4) |
| Informal only | 83 (9.8) | 70 (9.5) |
| Formal | 158 (18.6) | 134 (18.1) |
| **Maternal occupation** |  |  |
| Housewife | 709 (83.6) | 615 (83.2) |
| Farming and related | 28 (3.3) | 26 (3.5) |
| Trading and related | 96 (11.3) | 84 (11.4) |
| Other | 15 (1.8) | 14 (1.9) |
| **Maternal age** |  |  |
| 15-24 | 316 (37.3) | 275 (37.2) |
| 25-34 | 399 (47.1) | 350 (47.4) |
| 35-44 | 133 (15.7) | 114 (15.4) |
| **Water source** |  |  |
| River or Spring | 192 (22.7) | 166 (22.5) |
| Well | 134 (15.8) | 118 (16.0) |
| Pipe | 520 (61.3) | 453 (61.5) |
| **History of vaccination^∞^** |  |  |
| Vaccinated | 359 (42.5) | 307 (41.7) |
| Not vaccinated | 485 (57.5) | 429 (58.3) |
| **Household crowdedness*^∞∞^*** |  |  |
| 1-2 | 469 (55.3) | 399 (54.0) |
| 3-4 | 298 (35.1) | 266 (36.0) |
| 5-12 | 81 (9.6) | 74 (10.0) |
| **Intestinal parasitosis** |  |  |
| Yes | 218 (26.5) | 200 (27.1) |
| No | 604 (73.5) | 539 (72.9) |
| **Use of Antibiotics** |  |  |
| Yes | 115(14.2) | 105 (14.7) |
| No | 703(85.8) | 610 (85.3) |

*, ∞ measured at 2month, ∞∞ measured in person/household*
