# Supplementary material for: Effect of early and current Helicobacter pylori infection on the risk of anaemia in 6.5-year-old Ethiopian children
Source: BMC Infect Dis. 2015 Jul 14;15:270. doi: 10.1186/s12879-015-1012-y (PMC4501201; doi:10.1186/s12879-015-1012-y)
Supplement: Additional file 3: Table S2. — Haematological Parameters Mean (Standard deviation) and Anaemia Prevalence at Age 6.5 Years According to Child's H. pylori infection status from 3 to 6.5 Years of Age, Butajira Birth Cohort Study, Ethiopia. [file 12879_2015_1012_MOESM3_ESM.doc]

**Table S2.** Haematological Parameters Mean (Standard deviation) and Anaemia Prevalence at Age 6.5 Years According to Child's *H. pylori* infection status from 3 to 6.5 Years of Age, Butajira Birth Cohort Study, Ethiopia.

| **Variables** | **No. of**  **Children** | **Hb (g/dl)** | **MCH (pg)** | **MCHC (g/dl)** | **MCV (fl)** | **RWD (%)** | **Anemia prevalence^¥^**  **N ( %)** | **χ^2^ P value** ^¥¥¥^ |
| --- | --- | --- | --- | --- | --- | --- | --- | --- |
| **Exposure to *H. pylori* at age 3** |  |  |  |  |  |  |  |  |
| Yes | 212 | 11.8 (1.07) | 26.0 (2.07) | 31.6 (1.73) | 80.5 (4.48) | 14.5 (2.05 | 77 (36.3) |  |
| No | 300 | 11.7 (1.20) | 26.1 (2.41) | 31.7 (1.33) | 81.0 (5.25) | 14.6 (1.87) | 102 (34.5) | 0.32 |
| Total | 512* |  |  |  |  |  | 179 (34.9) |  |
| **Exposure to *H. pylori* at age 5** |  |  |  |  |  |  |  |  |
| Yes | 323 | 11.6 (1.10) | 26.0 (1.93) | 31.7 (1.37) | 80.9 (4.49) | 14.6 (2.04) | 128 (39.6) |  |
| No | 406 | 11.8 (1.12) | 26.3 (2.39) | 31.9 (1.54) | 81.2 (4.94) | 14.5 (1.73) | 124 (30.5) | 0.01 |
| Total | 729 |  |  |  |  |  | 252 (34.6) |  |
| **Exposure to *H. pylori* at age 6.5** |  |  |  |  |  |  |  |  |
| Yes | 73 | 11.7 (1.01) | 26.3 (1.88) | 31.7 (1.73) | 81.0 (4.20 | 14.6 (2.81 | 27 (37.0) |  |
| No | 666 | 11.8 (1.12) | 26.2 (1.24) | 31.8 (1.43) | 81.1 (4.82) | 14.6 (1.74) | 230 (34.5) | 0.38 |
| Total | 739 |  |  |  |  |  | 257 (34.8) |  |
| **Exposure to *H. pylori* up to age 6.5** ^¥¥^ |  |  |  |  |  |  |  |  |
| Never infected | 309 | 11.9 (1.13) | 26.4 (2.42) | 31.9 (1.17) | 80.6 (5.0) | 14.6 (1.7) | 88 (28.5) | 0.01 |
| Infected at any age up to age 6.5 | 430 | 11.7 (1.09) | 26.0 (2.02) | 31.6 (1.63) | 81.7 (4.5) | 14.5 (1.9) | 169 (39.3) |  |
| Total | 739 |  |  |  |  |  | 257 (34.8) |  |

¥Anaemia was defined according to WHO haemoglobin cutoffs: < 11.5 g/dL for children 5-11 years

¥¥  *H pylori infection at any age from 3-6.5 years*

¥¥¥ Chi square p- value was calculated between exposure to *H pylori* at different age (3-6.5) and prevalence of anaemia at age 6.5 years
